# Accumulation and attenuation of cardiovascular benefit with glucagon-like peptide 1 receptor agonists

**DOI:** 10.1136/bmjmed-2026-002704

**Published:** 2026-03-18

**Authors:** Jonathan A Batty, Tamara del Toro, Marlous Hall

**Affiliations:** 1 Hull York Medical School, York, England, UK; 2 Academic Cardiovascular Unit, James Cook University Hospital, South Tees NHS Foundation Trust, Middlesbrough, UK; 3 Leeds Institute for Data Analytics, University of Leeds, Leeds, West Yorkshire, UK; 4 Leeds Institute of Cardiovascular and Metabolic Medicine, University of Leeds, Leeds, West Yorkshire, UK

**Keywords:** Diabetes mellitus, Drug therapy, Cardiology, Myocardial infarction, Epidemiology

*Use of GLP-1 receptor agonists reduces the risk of cardiovascular events in people with type 2 diabetes, but these benefits may be reduced if treatment is interrupted; a linked study highlights the importance of target trial emulation in evaluating long term treatment strategies and informing future clinical and policy decisions, write **Batty and colleagues***

Glucagon-like peptide 1 receptor agonists (GLP-1RAs) improve glycaemic control,^1^ promote weight loss,^2^ delay progression of kidney disease,^3^ and reduce major adverse cardiovascular events (including myocardial infarction and stroke)^4^,^6^ in people with type 2 diabetes mellitus. The expanding indications of GLP-1RAs—including for individuals with obesity but who do not have diabetes—have driven rapid global uptake.^7^ However, discontinuation is common, with studies reporting that up to half of patients stop treatment with a GLP-1RA within the first year.^8 9^ Although previous evidence suggests that interruption or discontinuation of GLP-1RAs may diminish or reverse their beneficial cardiometabolic effects,^10^ the impact on major adverse cardiovascular events has remained uncertain.

To investigate this, Xie and colleagues conducted a target trial emulation analysis using routinely collected electronic health record data from the US Department of Veterans Affairs healthcare system (doi:10.1136/bmjmed-2025-002150).^11^ The target trial emulation framework provides a structured approach to mimicking a hypothetical randomised trial (the target trial) using large amounts of observational data according to a prespecified protocol.^12^ By aligning eligibility criteria, treatment strategies, follow-up, outcomes, and analytical methods with the target trial, the target trial emulation approach provides a structured way to answer a specific causal question from extensive healthcare data. If specified properly, target trial emulation can mitigate common biases in observational research, such as immortal time, misclassification, and selection bias,^13^ while offering the opportunity to efficiently investigate treatment effectiveness at whole population scale.

The authors assembled a retrospective cohort of 333 687 individuals with type 2 diabetes who had started treatment with either a GLP-1RA or a sulphonylurea (a class of glucose-lowering drug with no established cardioprotective benefit) between 2017 and 2023. Within the GLP-1RA arm, the researchers emulated a series of prespecified treatment strategies, reassigning treatment status every six months. This approach, known as sequential trial design, allowed the authors to investigate the complex structure of time-varying treatment regimens to capture continued GLP-1RA use, discontinuation, or interruption.^14 15^ Using inverse probability weighted marginal structural models to account for baseline and time-varying confounding, they estimated the three year cumulative incidence of major adverse cardiovascular events for 16 GLP-1RA treatment trajectories.

The key findings of this study suggest a duration dependent association between GLP-1RA usage and reduction in major adverse cardiovascular events. In total, 26% of new initiators of GLP-1RA discontinued treatment during follow-up, with two thirds of discontinuations occurring during the first year. Compared with those who received a sulphonylurea, a short duration (≤1.5 years) of GLP-1RA use was not associated with a significant reduction in major adverse cardiovascular events; however, continued GLP-1RA use for two years or longer was associated with significant risk reduction, with an 18% (95% confidence interval (CI) 15% to 22%) reduction in major adverse cardiovascular events for those who received continuous GLP-1RA treatment during the three year follow-up period. Interruption of GLP-1RA treatment was associated with an increased risk of major adverse cardiovascular events compared with continued GLP-1RA use: interruption at six months, one year, and two years was associated with 4% (95% CI 1% to 8%), 14% (9% to 18%), and 22% (16% to 27%) greater cumulative incidence of major adverse cardiovascular events, respectively.

These findings are plausible and in line with earlier randomised trial findings, which demonstrated consistent reductions in major adverse cardiovascular events in high risk populations with type 2 diabetes.^4 5^ Proposed mechanisms include favourable effects on glycaemic control, weight loss, lipid profiles, blood pressure, and systemic inflammation.^16 17^ Discontinuation has been observed to lead to weight regain and deterioration in cardiovascular risk factors,^10^ which may erode the benefits accrued. In their study,^11^ Xie and colleagues were not able interrogate the causal mechanisms through which discontinuation was associated with adverse cardiovascular outcomes.

This linked study^11^ highlights the value of applying the target trial emulation framework to routinely collected data to answer questions that cannot otherwise be reasonably examined in conventional randomised controlled trials. Randomising GLP-1RA de-prescription in type 2 diabetes would pose ethical concerns, given the proven benefits of GLP-1RAs in this population. Implementation of the target trial emulation framework enabled researchers to capitalise on inherent variation in clinical practice, allowing the impact of GLP-1RA discontinuation to be investigated in an ethical and efficient manner, at scale. While effective implementation of target trial emulation can be used to produce trial-like findings, their success depends on the appropriate use of causal inference methodology to minimise systematic biases seen in observational pharmacoepidemiology.^18^,^20^ The authors made several robust methodological choices, including the use of a sequential trial design and marginal structural models to mitigate the possible effect of time-varying confounding of treatment continuation on major adverse cardiovascular events. The scale of the Veterans Affairs healthcare dataset enabled well powered comparisons across multiple treatment trajectories for more than 330 000 individuals followed for three years—beyond what would be feasible in a randomised setting.

Several potential limitations of the reported study^11^ must also be considered. Although the authors reported their predefined protocol at the time of publication (complete with eligibility criteria, treatment strategies, outcomes, and estimands), advanced registration of a full study protocol is considered best practice. Given the non-randomised nature of this study, important unmeasured differences may remain between individuals who continued and discontinued treatment. The authors were unable to explore reasons for high discontinuation rates observed or to evaluate differences between individual GLP-1RA agents. The cohort under investigation included mostly older men with diabetes, so further work is required to substantiate these findings for younger people, female groups, and those who do not have diabetes but who use GLP-1RAs for obesity.

As GLP-1RAs move from specialist diabetes clinics into wider clinical practice, greater clinical attention must be given to long term treatment trajectories and improvement of treatment continuity, to maintain the cardioprotective effects seen with GLP-1RAs. Future research should focus on the generalisability of these findings, better understanding drivers of discontinuation, and evaluation of strategies that support sustained GLP-1RA use in people with type 2 diabetes. These approaches may include structured follow-up during treatment initiation, improved management of gastrointestinal side effects, and the development of novel agents with improved side effect profiles. Leveraging population scale data to monitor and optimise long term treatment trajectories will be central to translating the cardiovascular promise of GLP-1RAs into durable real world benefit.
